# Supplementary figures and images for: Modular UBE2H-CTLH E2-E3 complexes regulate erythroid maturation
Source: eLife. 2022 Dec 2;11:e77937. doi: 10.7554/eLife.77937 (PMC9718529; doi:10.7554/eLife.77937)

Figure 1D

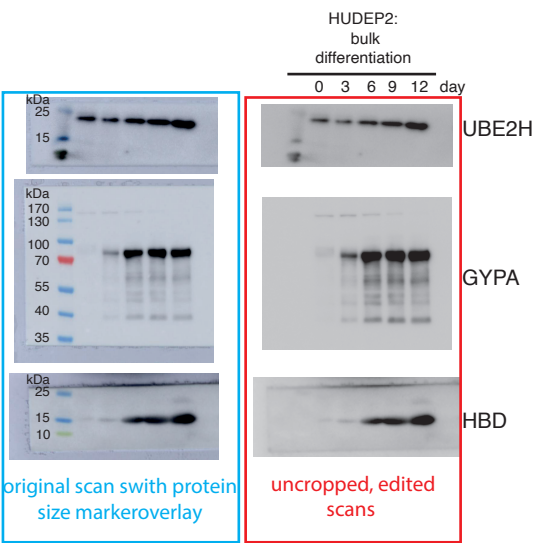

Figure 1E

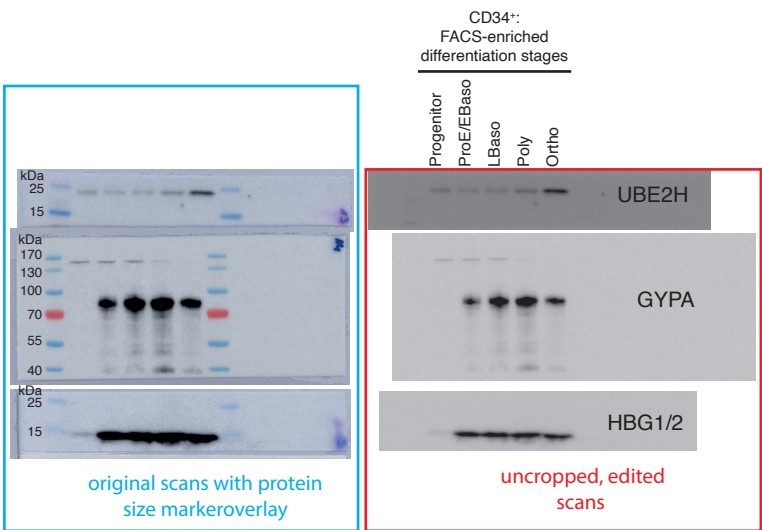

Figure 1–source data 1

Supplement: Figure 1—source data 1. [file elife-77937-fig1-data1.pdf]

Figure 2C

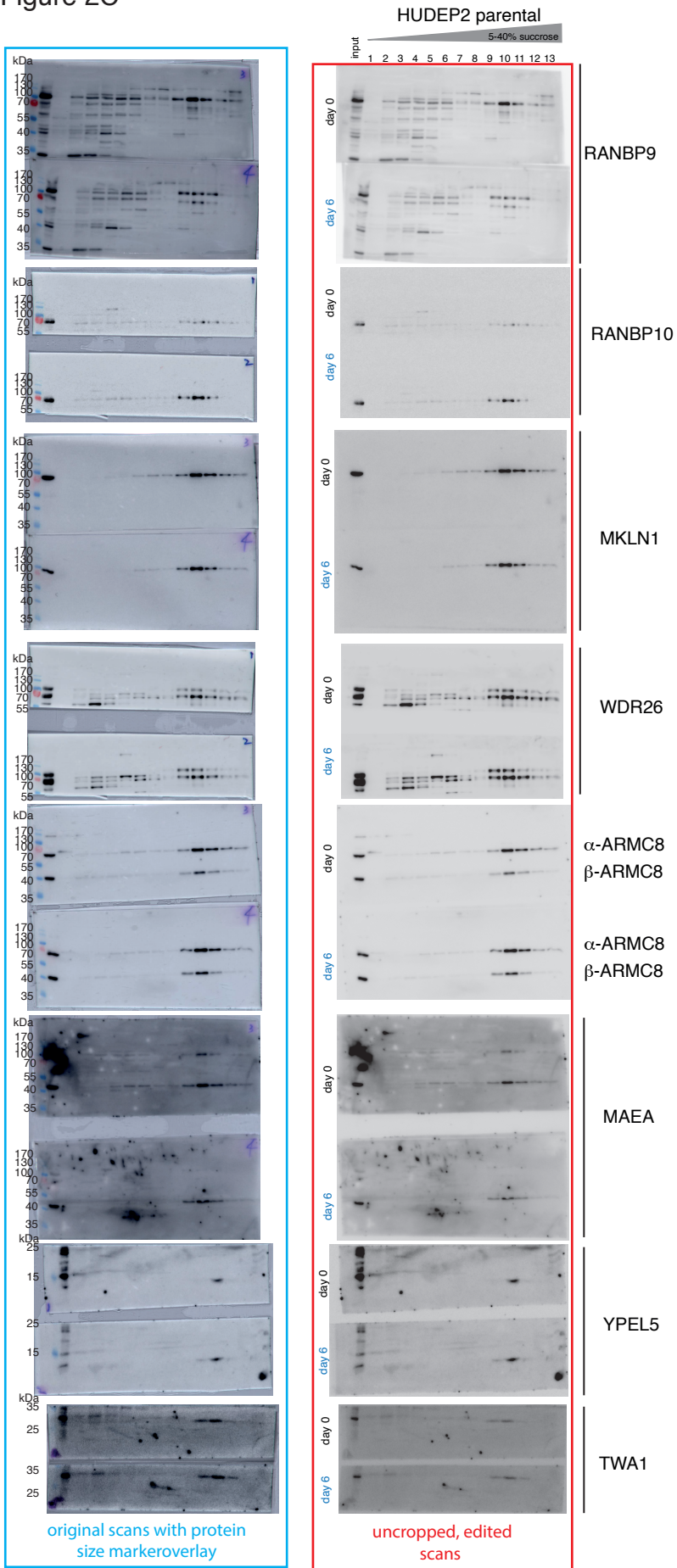

### Figure 2—source data 1

Supplement: Figure 2—source data 1. [file elife-77937-fig2-data1.pdf]

Figure 2E

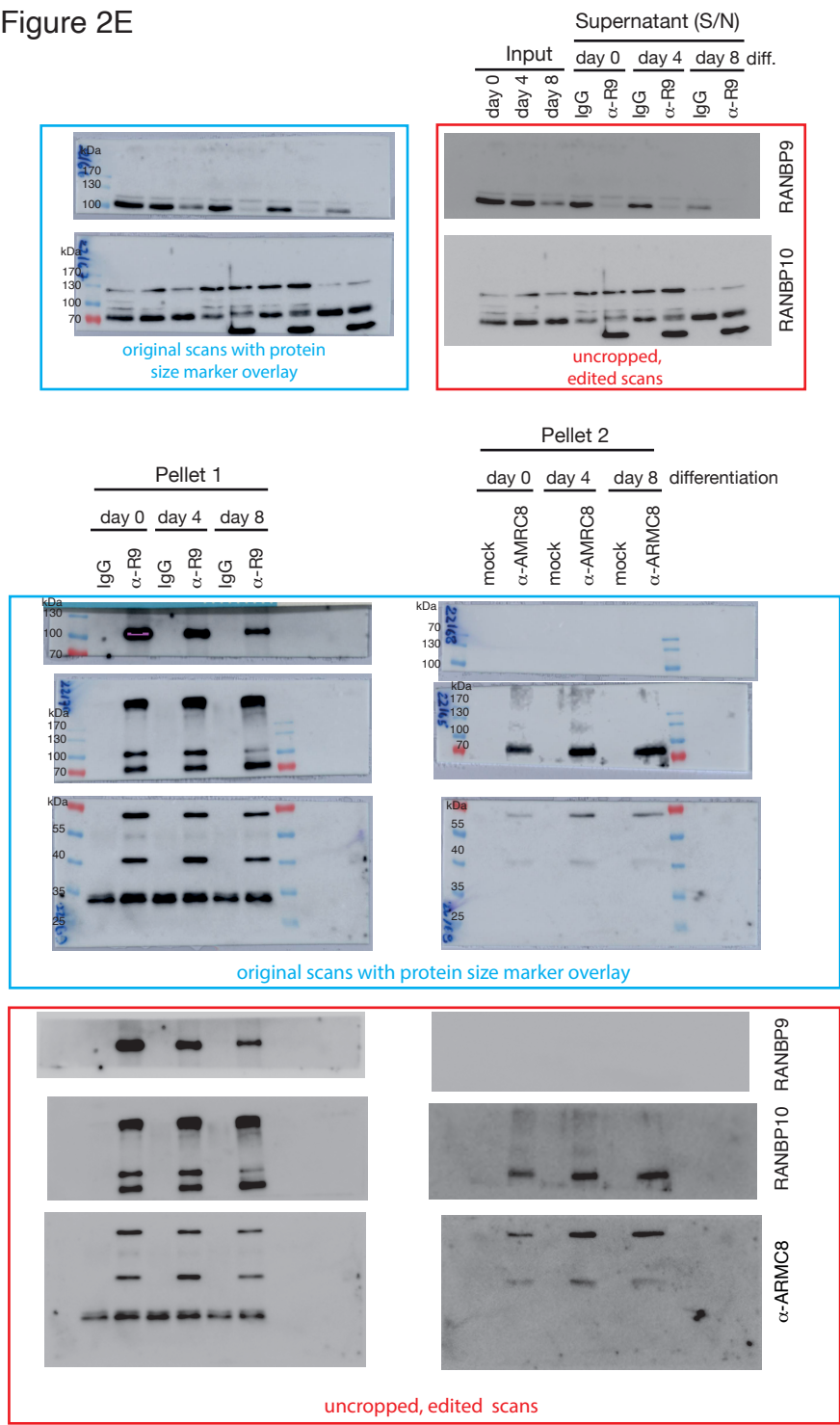

Figure 2F

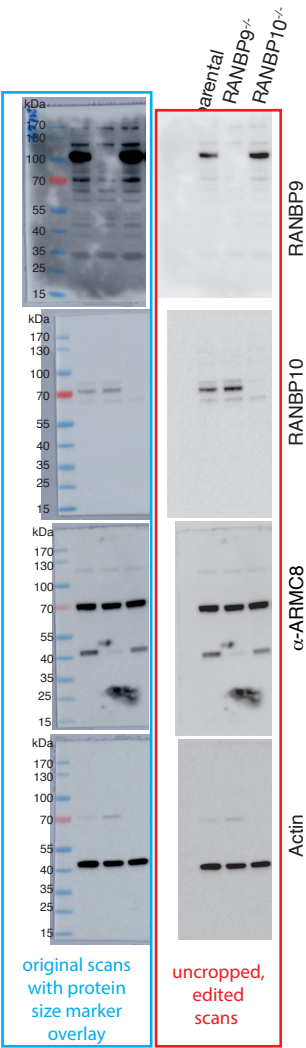

Figure 2–source data 2

Supplement: Figure 2—source data 2. [file elife-77937-fig2-data2.pdf]

Figure 2G

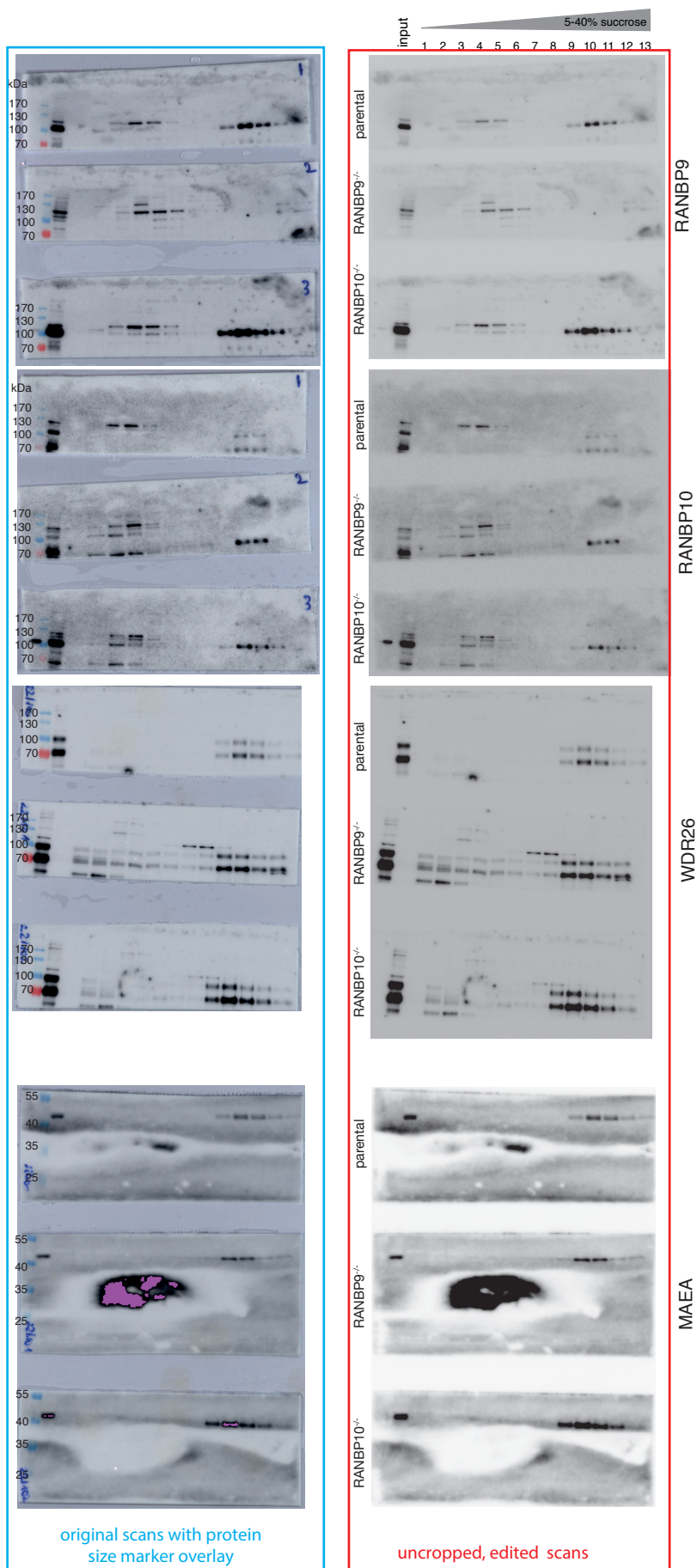

Figure 2–source data 3

Supplement: Figure 2—source data 3. [file elife-77937-fig2-data3.pdf]

Figure 3A

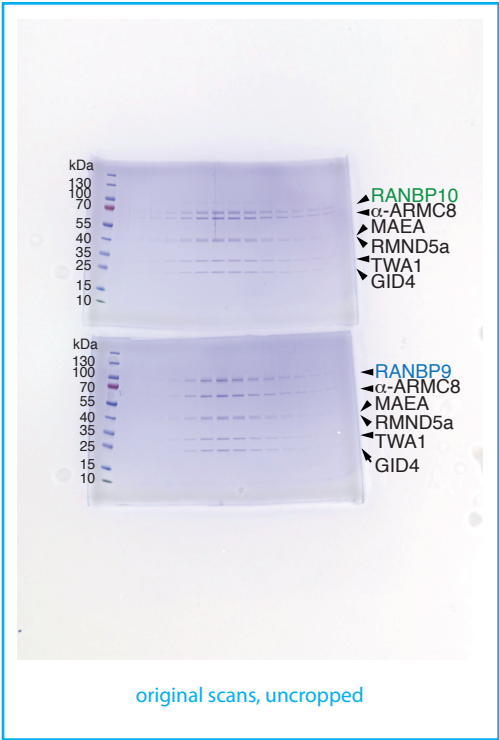

Figure 3D

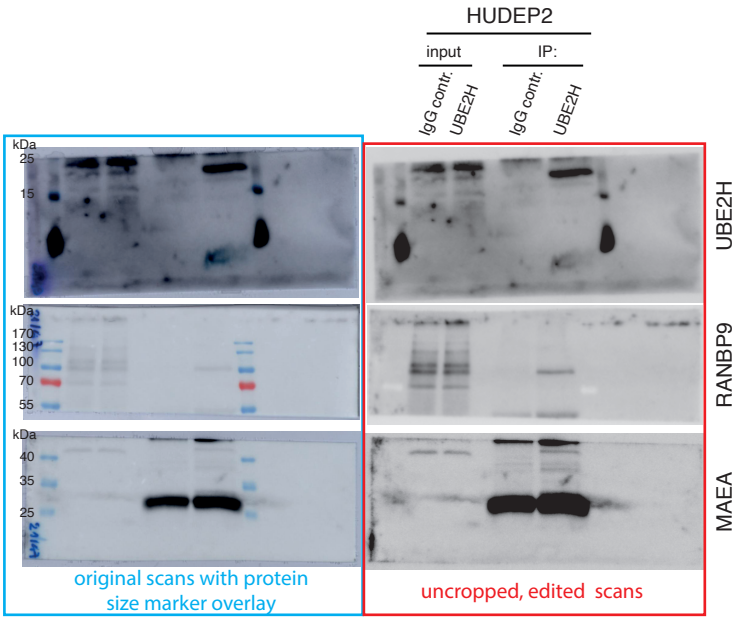

Figure 3E

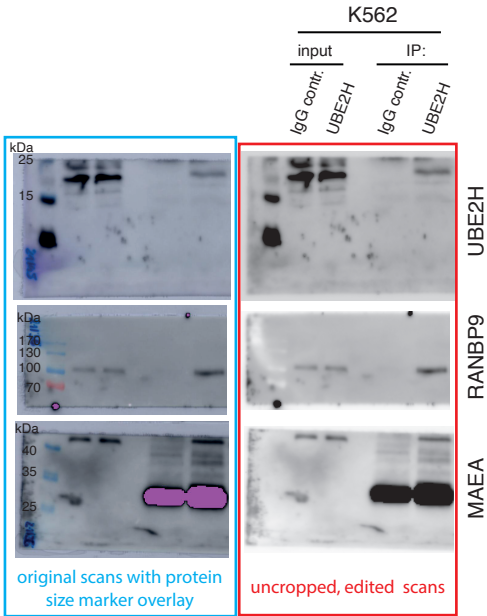

Figure 3F

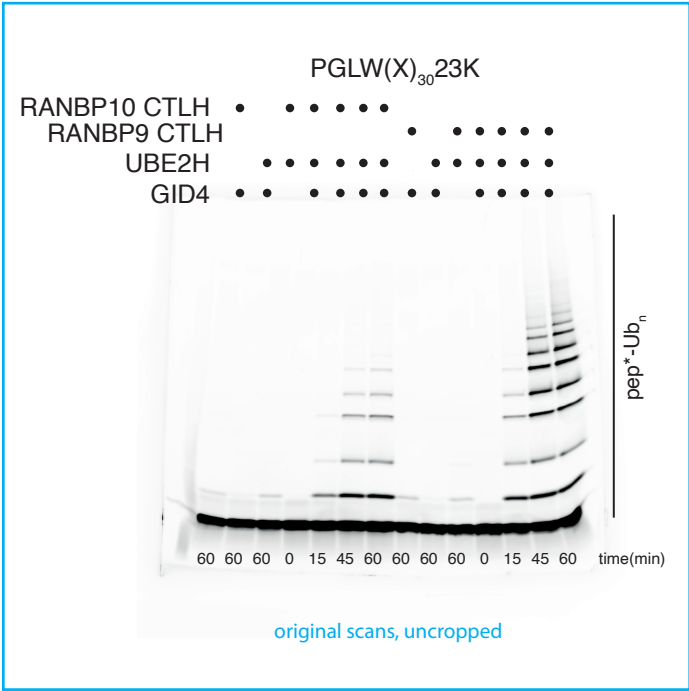

Figure 3—source data 1

Supplement: Figure 3—source data 1. [file elife-77937-fig3-data1.pdf]

Figure 4A

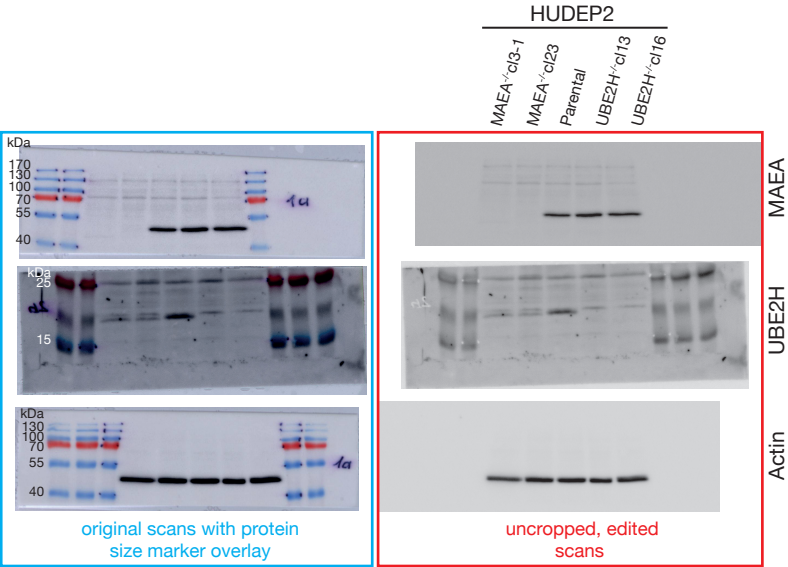

Figure 4–source data 1

Supplement: Figure 4—source data 1. [file elife-77937-fig4-data1.pdf]

Figure 1–figure supplement 1C

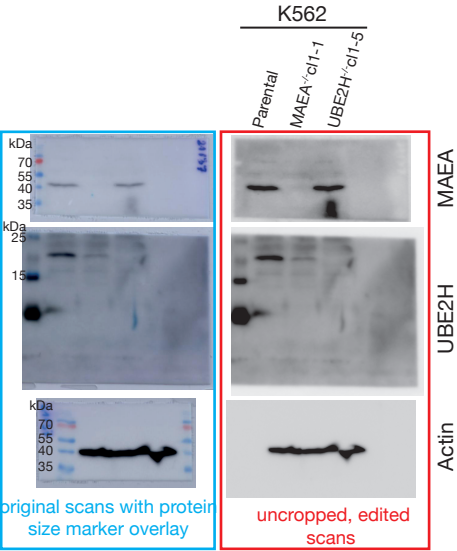

Figure 4–figure supplement 1–source data 1

Supplement: Figure 4—figure supplement 1—source data 1. [file elife-77937-fig4-figsupp1-data1.pdf]

Figure 4–figure supplement 2C

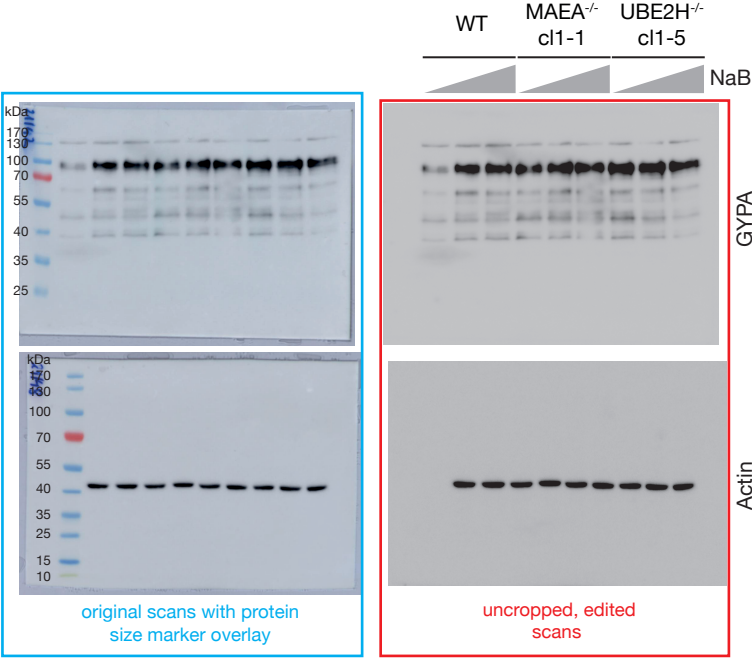

Figure 4–figure supplement 2–source data 1

Supplement: Figure 4—figure supplement 2—source data 1. [file elife-77937-fig4-figsupp2-data1.pdf]

Figure 5A

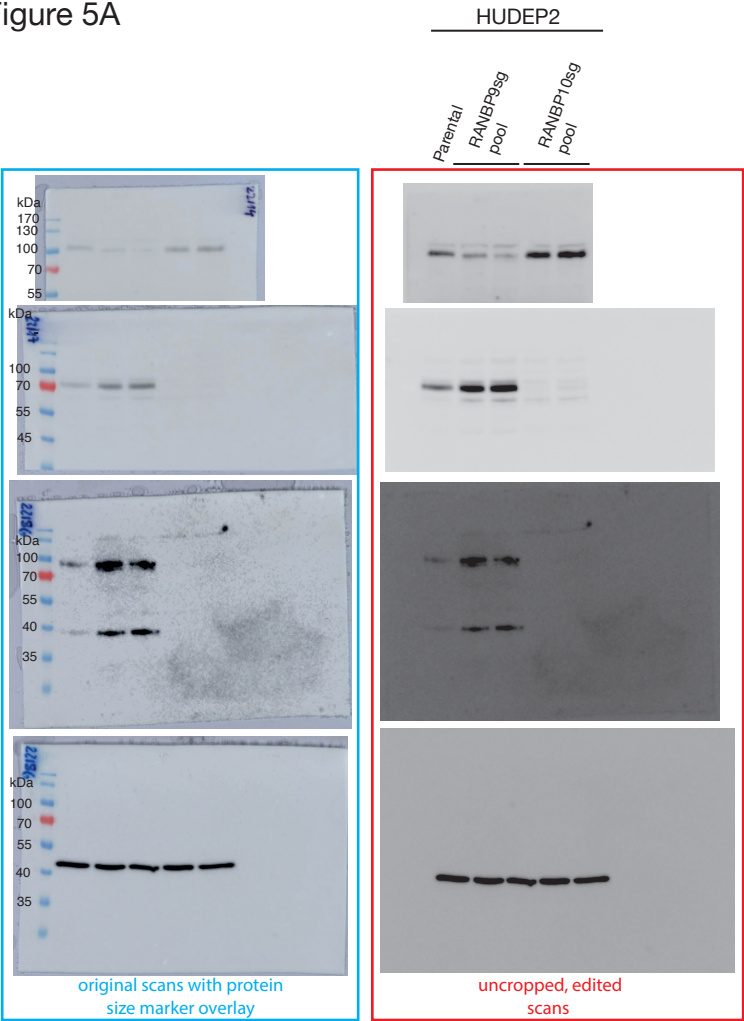

Figure 5C

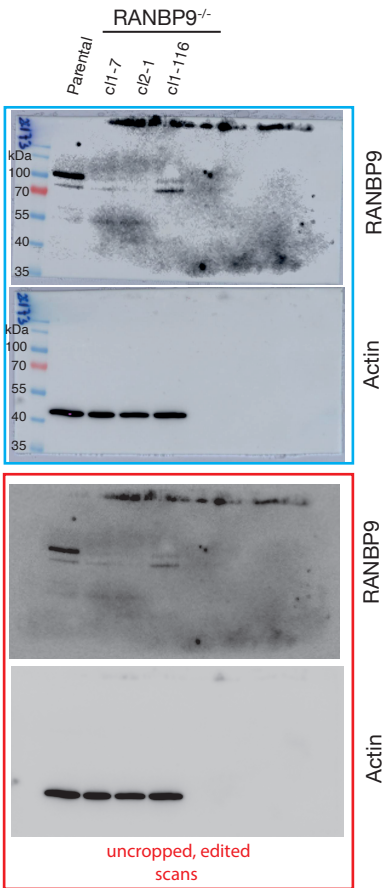

Figure 5D

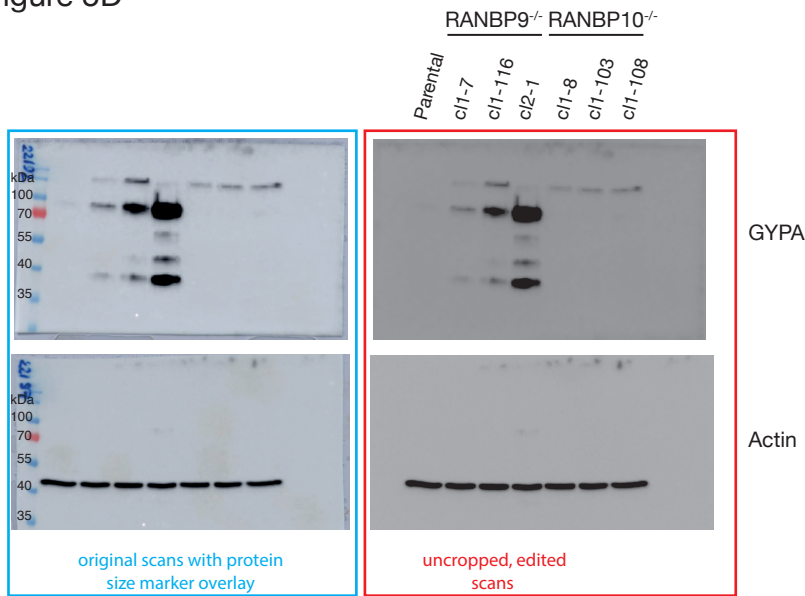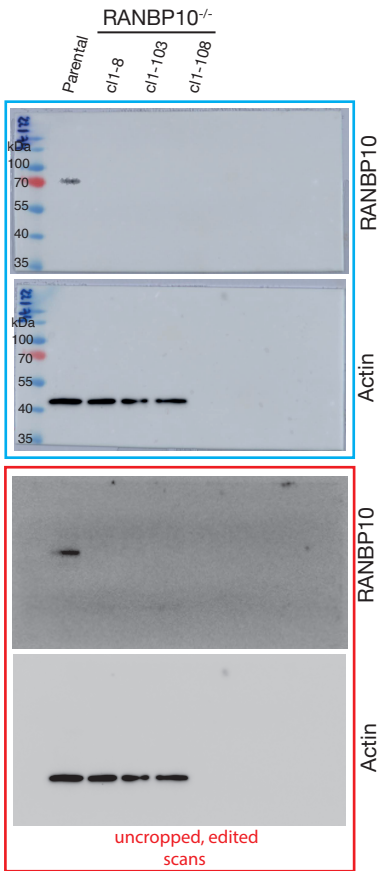

Figure 5-source data 1

Supplement: Figure 5—source data 1. [file elife-77937-fig5-data1.pdf]

Figure 6A

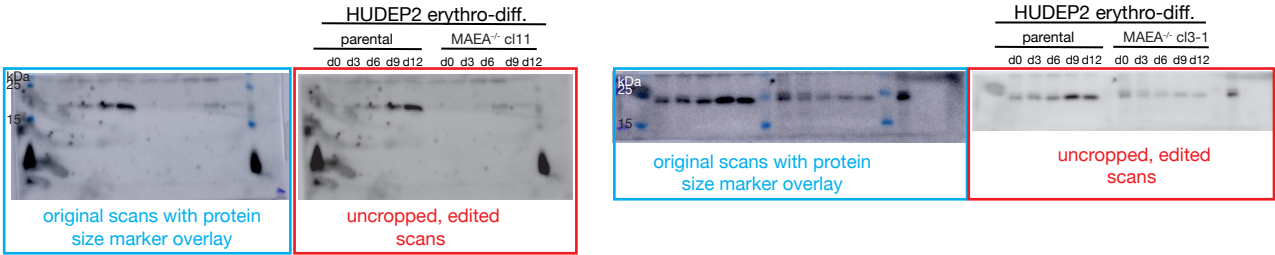

Figure 6D

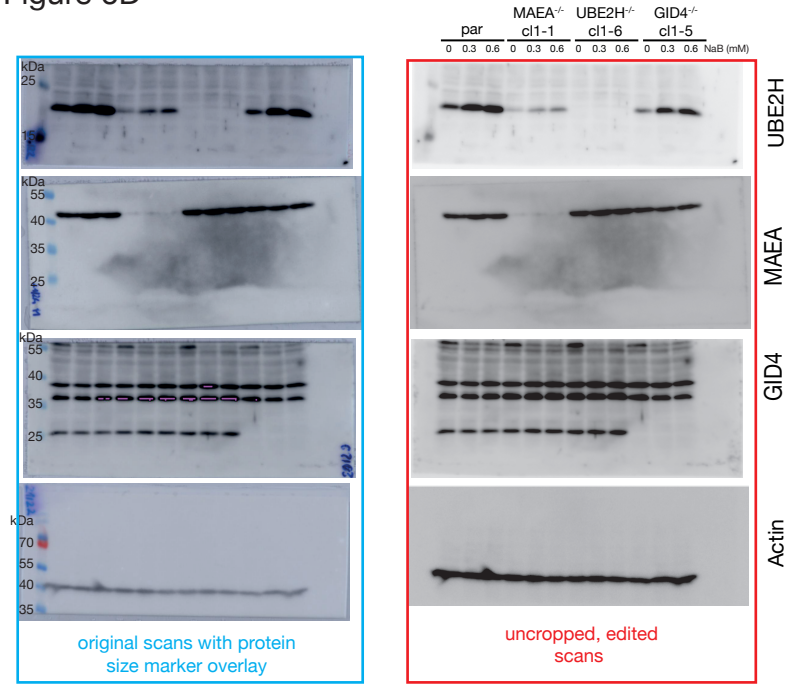

Figure 6E

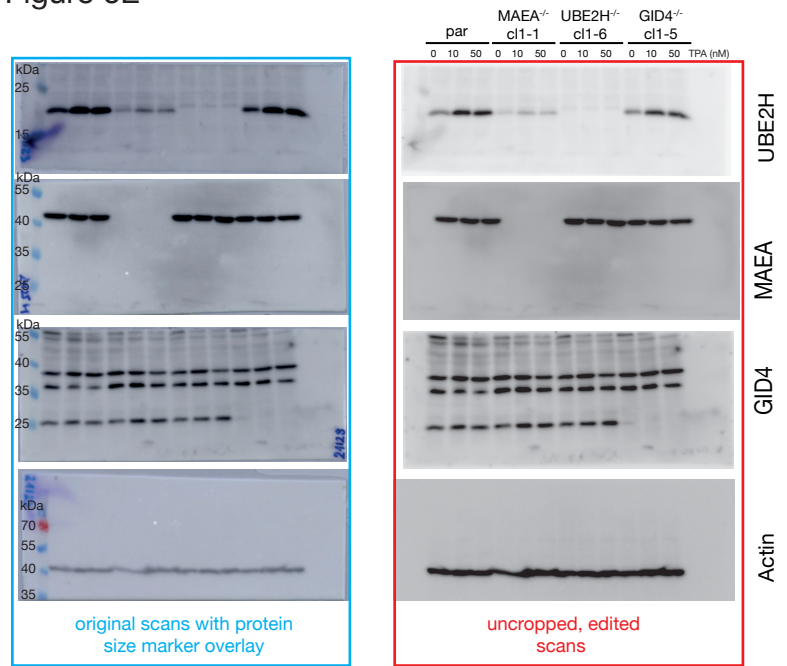

Figure 6—source data 1

Supplement: Figure 6—source data 1. [file elife-77937-fig6-data1.pdf]

Figure 6G

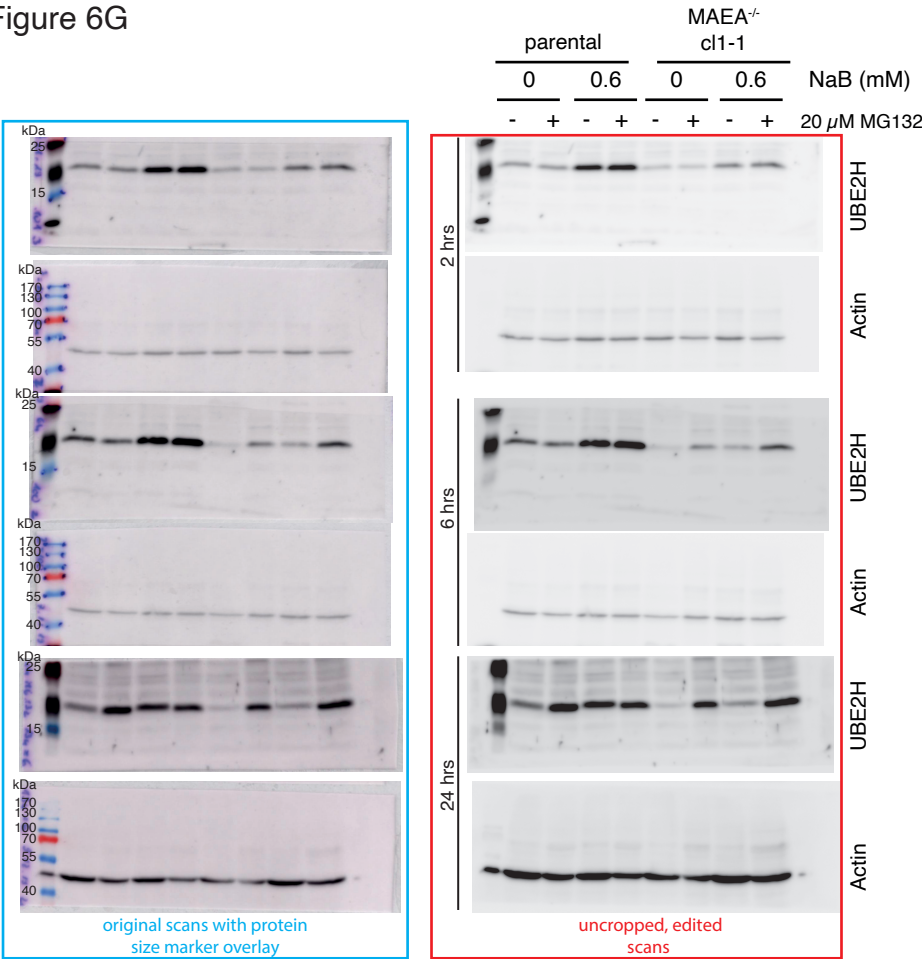

Figure 6I

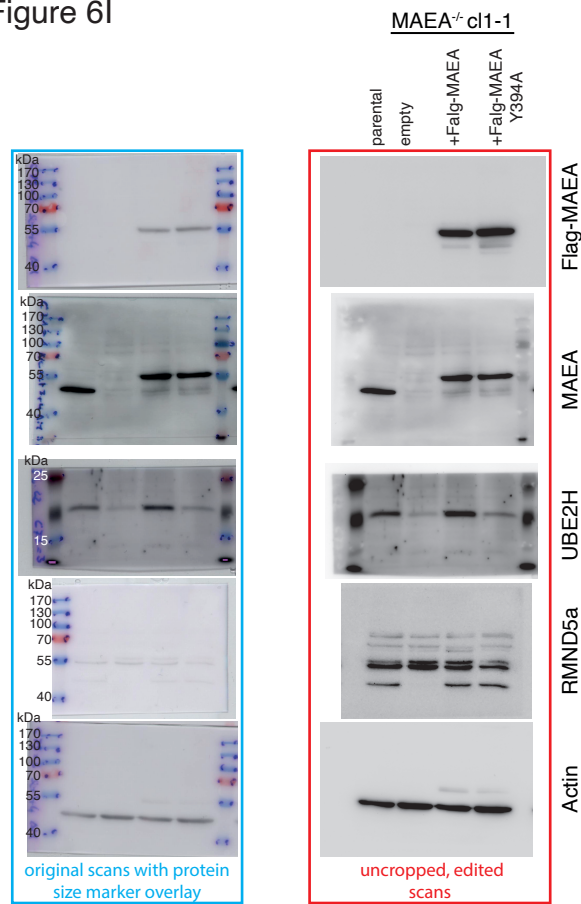

Figure 6K

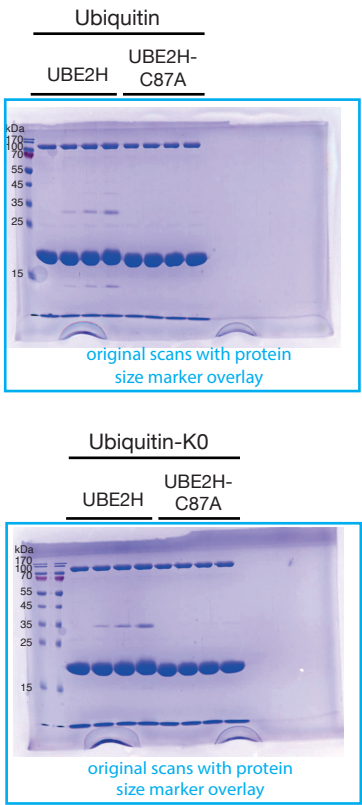

Figure 6—source data 2

Supplement: Figure 6—source data 2. [file elife-77937-fig6-data2.pdf]
