# Supplementary material for: Modular UBE2H-CTLH E2-E3 complexes regulate erythroid maturation
Source: eLife. 2022 Dec 2;11:e77937. doi: 10.7554/eLife.77937 (PMC9718529; doi:10.7554/eLife.77937)
Supplement: Figure 6—figure supplement 1—source data 1. [file elife-77937-fig6-figsupp1-data1.pdf]

Figure 6–figure supplement 1A

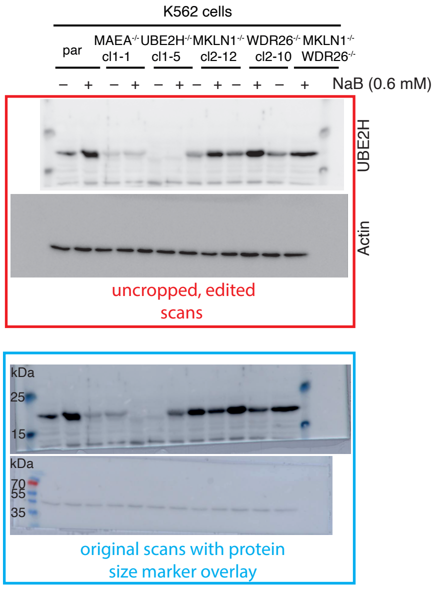

Figure 6–figure supplement 1B

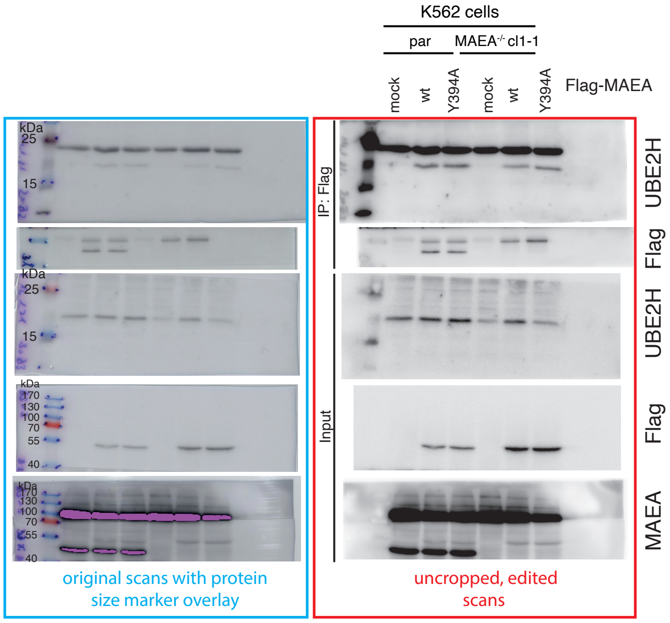

Figure 6–figure supplement 1C

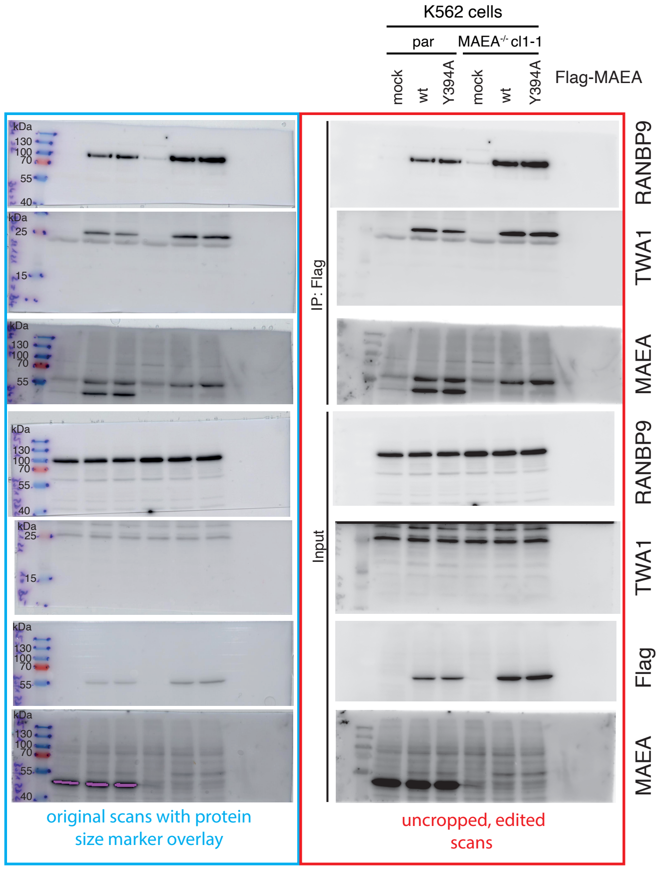

Figure 6–figure supplement 1–source data 1
